# Prediction of humeral shaft fracture healing using the Radiographic Union Score for HUmeral Fractures (RUSHU)

**DOI:** 10.1302/2633-1462.511.BJO-2024-0134.R1

**Published:** 2024-11-04

**Authors:** Cyrill Suter, Henrik Mattila, Thomas Ibounig, Bakir O. Sumrein, Antti Launonen, Teppo L. N. Järvinen, Tuomas Lähdeoja, Lasse Rämö

**Affiliations:** 1 Finnish Centre for Evidence-Based Orthopaedics (FICEBO), Department of Orthopaedics and Traumatology, University of Helsinki and Helsinki University Hospital, Helsinki, Finland; 2 Department of Orthopaedics and Traumatology, University of Tampere and Tampere University Hospital, Wellbeing services county of Pirkanmaa, Tampere, Finland

**Keywords:** Traumatology, Humeral shaft, Fracture, Humerus, Union, RUSHU, Score, Nonunion, Prediction, humeral shaft fractures, Humeral Fractures, fracture nonunion, intraclass correlation coefficients (ICCs), radiological scoring, surgical treatment, Radiographs, FISH trial, randomized controlled trials, fracture healing

## Abstract

**Aims:**

Though most humeral shaft fractures heal nonoperatively, up to one-third may lead to nonunion with inferior outcomes. The Radiographic Union Score for HUmeral Fractures (RUSHU) was created to identify high-risk patients for nonunion. Our study evaluated the RUSHU’s prognostic performance at six and 12 weeks in discriminating nonunion within a significantly larger cohort than before.

**Methods:**

Our study included 226 nonoperatively treated humeral shaft fractures. We evaluated the interobserver reliability and intraobserver reproducibility of RUSHU scoring using intraclass correlation coefficients (ICCs). Additionally, we determined the optimal cut-off thresholds for predicting nonunion using the receiver operating characteristic (ROC) method.

**Results:**

The RUSHU demonstrated good interobserver reliability with an ICC of 0.78 (95% CI 0.72 to 0.83) at six weeks and 0.77 (95% CI 0.71 to 0.82) at 12 weeks. Intraobserver reproducibility was good or excellent for all analyses. Area under the curve in the ROC analysis was 0.83 (95% CI 0.77 to 0.88) at six weeks and 0.89 (95% CI 0.84 to 0.93) at 12 weeks, indicating excellent discrimination. The optimal cut-off values for predicting nonunion were ≤ eight points at six weeks and ≤ nine points at 12 weeks, providing the best specificity-sensitivity trade-off.

**Conclusion:**

The RUSHU proves to be a reliable and reproducible radiological scoring system that aids in identifying patients at risk of nonunion at both six and 12 weeks post-injury during non-surgical treatment of humeral shaft fractures. The statistically optimal cut-off values for predicting nonunion are ≤ eight at six weeks and ≤ nine points at 12 weeks post-injury.

## Introduction

Functional bracing has traditionally been the mainstay in the treatment of humeral shaft fractures.^1^ Two recent randomized controlled trials (RCTs) that compared functional bracing to surgery did not find any clinically relevant differences between the treatment strategies at one year.^2,3^ However, up to one-third of the patients randomized to functional bracing experienced healing problems and required secondary surgery, with inferior functional outcomes even though the fractures eventually healed successfully.^4^ The prevalence of nonunion following nonoperative treatment for humeral shaft fractures has exhibited consistent figures across diverse populations, ranging from 10% to 33%.^3-9^

Given the substantial incidence of this complication, enhancing our capacity to promptly identify patients at higher risk of developing fracture nonunion would prove beneficial. The initial algorithms for predicting fracture healing or nonunion, which relied on radiological scores derived from a systematic evaluation of follow-up radiographs, were developed and subsequently validated for femur and tibia shaft fractures.^10,11^ The Radiographic Union Score for HUmeral Fractures (RUSHU)^12^ was adapted from these in 2019. The original study used radiographs taken six weeks post-injury to identify humeral shaft fractures prone to nonunion.^12^ The RUSHU scoring system has shown promising results in predicting fracture healing, exhibiting good inter- and intraobserver reliability. To our knowledge, these findings have been limited to relatively small cohorts, ranging from 32 to 92 patients.^13,14^ Our objective was to validate the RUSHU in a considerably larger cohort and to assess its prognostic performance at both six- and 12-week follow-up.

## Methods

This study was conducted at the Helsinki University Hospital, with approval from the local Institutional Review Board (Dno HUS/146/2023).

### Derivation of study cohort

Our preliminary cohort consisted of: 1) all non-pathological and non-periprosthetic humeral shaft fracture patients aged 18 years or older treated in our unit (Helsinki University Hospital) between January 2006 and December 2016, identified through a retrospective analysis;^15^ and 2) the patients participating in the Finnish Shaft of the Humerus (FISH) trial, a RCT comparing effectiveness of surgery and non-surgical treatment of humeral shaft fractures (ClinicalTrials.gov, NCT01719887). The FISH trial was executed in two large trauma centres (Helsinki University Hospital and Tampere University Hospital) in Finland between 2012 and 2018.^16^

From this preliminary cohort of 1,000 individuals with a humeral shaft fracture, we included the patients whose treatment was nonoperative, and the following information was available: 1) anteroposterior and lateral radiographs at six- or 12-week follow-up; 2) known fracture healing outcome (union or nonunion); and 3) no conversion to surgery before the 12-week follow-up. Radiographs taken within ten days of the six-week and 20 days of the 12-week post-injury dates were accepted.

**Fig. 1:**
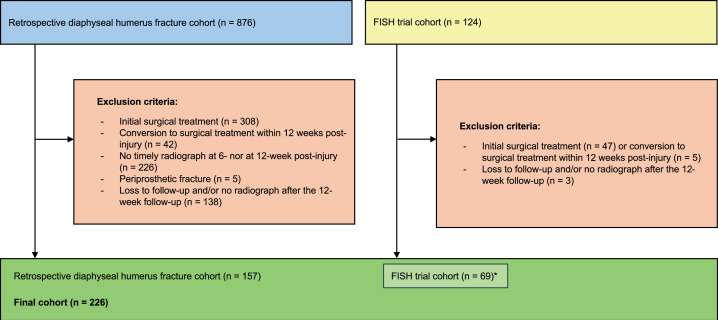
Flowchart of the study (*Finnish Shaft of the Humerus (FISH) trial participants were included in a pre-planned subgroup sensitivity analysis and in the primary analysis).

To ascertain treatment outcome, we required at least one additional radiograph after the 12-week follow-up, which either verified a fracture union or an established nonunion, or for patients who underwent surgery after the 12-week follow-up, a verification of nonunion in the surgical notes. The application of these criteria resulted in a final cohort of 226 cases with verified fracture healing outcome (Figure 1).

### Assessment of fracture healing status

Radiographs were assessed independently by two readers (CS, HM) blinded to the fracture healing outcome. For the blinded assessment of radiographs, the six- and 12-week follow-up radiographs were extracted from the Picture Archiving and Communication System (Siemens, Germany) and assigned a randomly generated ID label. Prior to the actual scoring, we conducted a pre-validation of the RUSHU in 20 randomly selected cases. The objective of pre-validation was to familiarize the readers with the RUSHU and harmonize its application. After pre-validation of 20 cases, we discussed the pre-validation findings in plenum.

**Fig. 2:**
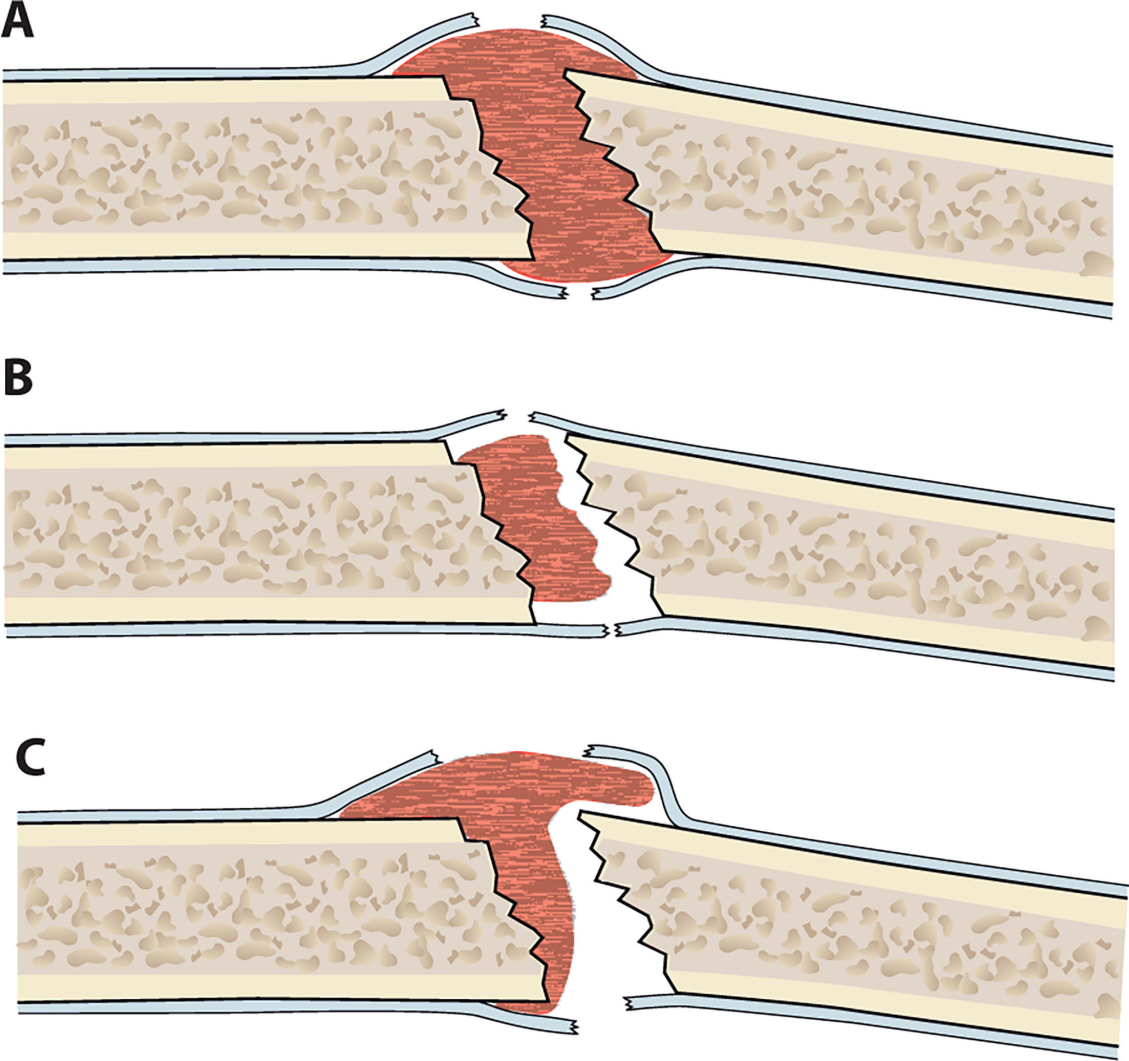
Illustration of (A) bridging callus on both cortices, (B) non-bridging callus on both cortices, and (C) our intermediate form “M” for Maybe on the upper cortices, where callus extends beyond a virtual line drawn between proximal and distal cortices and callus envelopes the exposed cortices, but the callus is not bridging, and non-bridging callus on the lower cortices.

The final scoring was carried out using the criteria outlined in the original study by Oliver et al.^12^ Briefly, all four cortices from anterior-posterior and lateral radiographs were assessed and graded according to healing status, as follows: no visible callus = one point; non-bridging callus = two points; and bridging callus = three points. Accordingly, a total score of each radiograph ranged from four to 12 points. We encountered some ambiguity in the original publication regarding the definition of “bridging of callus”.^12^ According to the initial scoring, if the callus extends beyond a virtual line drawn between proximal and distal cortices, or if it envelops the exposed cortices, it merits three points. In situations where the criteria were met, but the callus did not make direct contact with the corresponding cortex, we designated such cases with an “M” for maybe (Figure 2). In our primary analysis, we assigned three points to these cases, aligning with the original study’s suggestion. However, we also performed a sensitivity analysis wherein “M”-cases were given two points. To ensure consistency, all cases with a difference of one or more RUSHU points underwent re-evaluation by both the original readers and a minimum of two other senior authors (LR and TI or TL) in a consensus meeting.

The STARD 2015 guideline was used for the reporting of this study.^17^

### Statistical analysis

Statistical analysis was carried out with SPSS v. 29.0.1.0 (IBM, USA). The normality of continuous variables was assessed using the Shapiro-Wilk test. Mann-Whitney U test was used for analyzing non-parametric continuous data (age at the time of injury). The relationship between categorical variables was assessed using a chi-squared test or two-sided Fisher’s exact test if the expected count was less than five for more than 20% of the cells. Interobserver reliability and intraobserver reproducibility were assessed using the intraclass correlation coefficient (ICC) with a two-way mixed-effects model, and reported using single measures ICC with a 95% CI. The ICCs were interpreted as follows: values less than 0.5 indicate poor reliability, 0.5 to 0.75 indicates moderate reliability, 0.75 to 0.9 is indicative of good reliability, and values greater than 0.9 indicate excellent reliability.^18^

We conducted two prespecified sensitivity analyses to assess: 1) the M-cases with two instead of three points; and 2) the prognostic performance of the RUSHU in the FISH trial cohort only. The latter sensitivity analysis was prompted by an observation that in the larger retrospective cohort (n = 876), patients who went on to an uneventful union but did not have follow-up radiographs ascertaining the union beyond 12 weeks were missing, thus artificially inflating the nonunion rate of the cohort. The analysis of the FISH trial cohort derived from a prospective controlled trial provided a more robust follow-up data for assessing the prognostic performance of the RUSHU.

Radiographs of the retrospective cohort were scored once for interobserver reliability analysis. The FISH trial cohort was additionally assessed a second time after three months for intraobserver reproducibility analysis.

The optimal RUSHU cut-off to predict nonunion was determined by a receiver operating characteristic (ROC) analysis using the closest point to top left corner method.^19^ The discrimination capability (area under curve (AUC)) is classified as follows: 0.7 to 0.8 is considered as acceptable, 0.8 to 0.9 excellent, and more than 0.9 outstanding.^20^ The threshold for significance was set at level 0.05 with two-sided testing.

## Results

### Baseline characteristics

A total of 226 cases were included in this study (157 from the retrospective cohort and 69 from the FISH trial; Figure 1). The baseline demographics of the patients and injuries are presented in Table I. Radiographs were available for 204 cases at six weeks and 206 cases at 12 weeks. In the total cohort, the overall nonunion rate was 41% (92/226). Among these, 70% (64/92) underwent surgery, performed at a mean of 205 days (79 to 530) post-injury.

**Table I.** Baseline demographic data.

Variable	Total (n = 226)	Union (n = 134)	Nonunion (n = 92)	p-value
Sex, female:male, n (%)	115:111 (51:49)	65:69 (49:51)	50:42 (54:46)	0.388*
Mean age, yrs (range)	57 (19 to 91)	53 (19 to 91)	62 (21 to 91)	< 0.001†§
**Smoker, n (%)**				0.174*
Smoker	65 (29)	34 (25)	31 (34)	
Non-smoker	161 (71)	100 (75)	61 (66)	
**Displacement, n (%)**				< 0.001‡§
Not displaced	6 (3)	5 (4)	1 (1)	
Displaced under a shaft width	202 (89)	126 (94)	76 (83)	
Displaced over a shaft width	18 (8)	3 (2)	15 (16)	
**AO classification, n (%)**				0.200*
A (simple)	153 (68)	88 (65)	65 (71)	
B (wedge fragment)	54 (24)	37 (28)	17 (18)	
C (segmental)	19 (8)	9 (7)	10 (11)	
**Location, n (%)**				< 0.001*§
Proximal shaft	38 (17)	14 (10)	24 (26)	
Mid-shaft	175 (77)	107 (80)	68 (74)	
Distal shaft	13 (6)	13 (10)	0 (0)	

* Chi-squared test.

† Mann-Whitney U test.

‡ Fisher’s exact test.

§ Statistically significant.

### Primary results

The intraobserver ICC for reader one was 0.94 (95% CI 0.91 to 0.96) at six weeks and 0.98 (95% CI 0.97 to 0.99) at 12 weeks, indicating excellent reproducibility. For reader two, ICCs were 0.87 (95% CI 0.79 to 0.92) at six weeks and 0.93 (95% CI 0.88 to 0.96) at 12 weeks, indicating good and excellent reproducibility.

The interobserver ICC was 0.78 (95% CI 0.72 to 0.83) at six weeks and 0.77 (95% CI 0.71 to 0.82) at 12 weeks, indicating good reliability at both timepoints. Further reliability analyses of individual cortex scores showed no significant differences regarding which cortex was scored most or least reliably (Table II).

**Table II.** The interobserver reliability of individual cortex scores and total RUSHU score, and intraobserver reproducibility at six and 12 weeks.

Cortex	Interobserver ICC, 6 weeks (95% CI)	Interobserver ICC, 12 weeks (95% CI)
Lateral	0.65 (0.56 to 0.72)	0.68 (0.59 to 0.74)
Medial	0.68 (0.60 to 0.75)	0.58 (0.48 to 0.66)
Anterior	0.62 (0.53 to 0.70)	0.63 (0.54 to 0.71)
Posterior	0.64 (0.55 to 0.71)	0.70 (0.63 to 0.77)
Total RUSHU score	0.78 (0.72 to 0.83)	0.77 (0.71 to 0.82)
Intraobserver ICC reader 1	0.94 (0.91 to 0.96)	0.98 (0.97 to 0.99)
Intraobserver ICC reader 2	0.87 (0.79 to 0.92)	0.93 (0.88 to 0.96)

ICC, intraclass correlation coefficient; RUSHU, Radiographic Union Score for HUmeral Fractures.

**Fig. 3:**
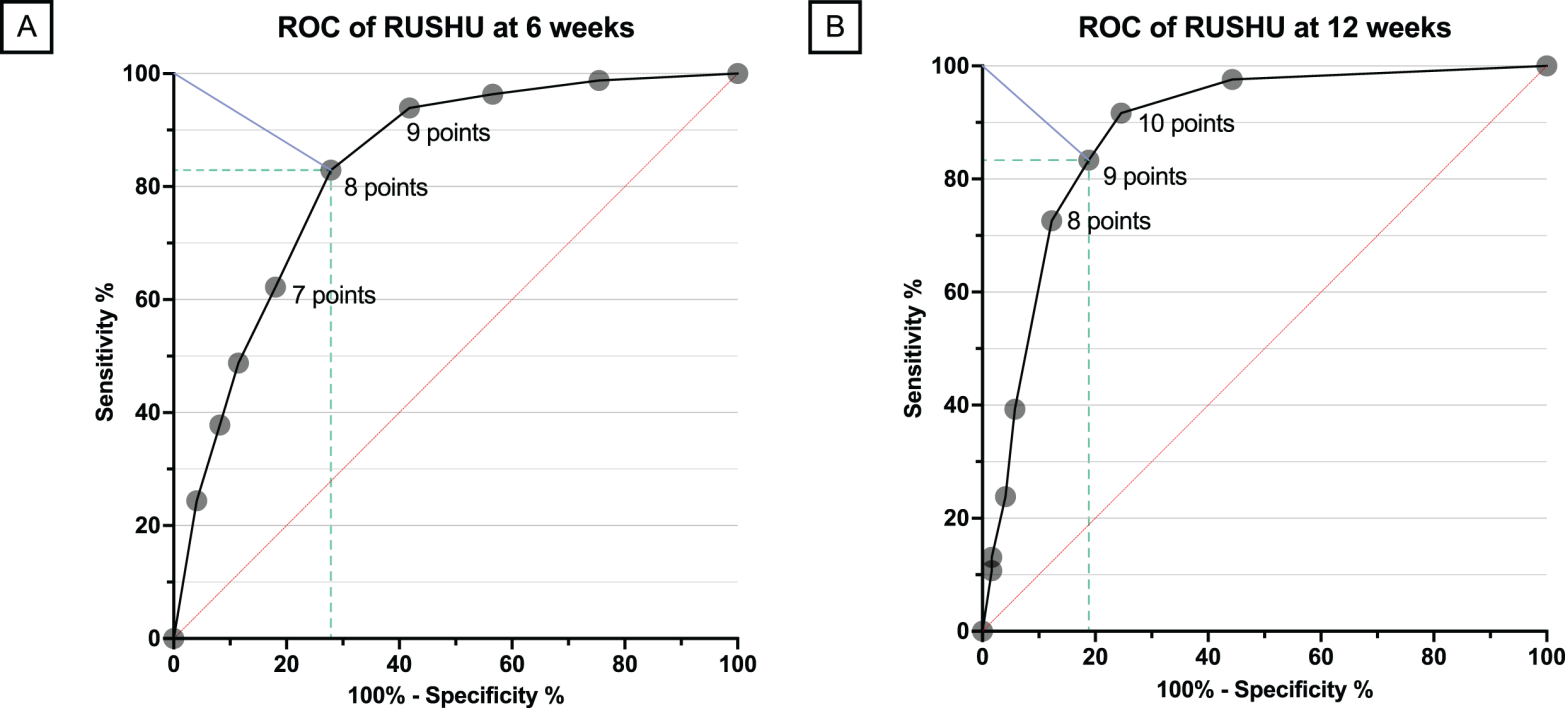
Receiver operating characteristic (ROC) curve for the Radiographic Union Score for HUmeral Fractures (RUSHU) at (A) six weeks (n = 204) and (B) 12 weeks (n = 206).

ROC curves for the RUSHU yielded an AUC of 0.83 (95% CI 0.78 to 0.89) at six weeks and 0.89 (95% CI 0.84 to 0.93) at 12 weeks, indicating excellent discrimination. Using the closest point to the left corner method, cut-off values of ≤ eight and ≤ nine points provided the best specificity-sensitivity trade-off for predicting nonunion at six- and 12-week follow-up, respectively (Figure 3).

**Fig. 4:**
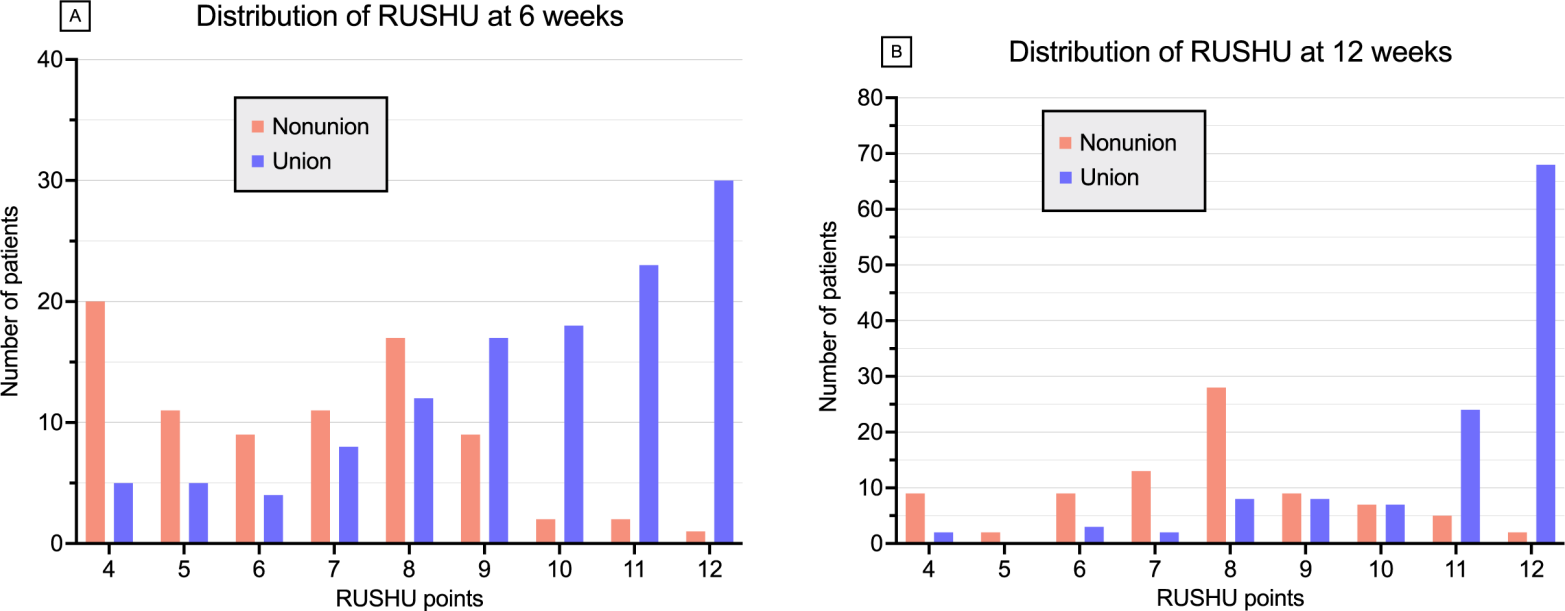
Distribution of the Radiographic Union Score for HUmeral Fractures (RUSHU) at a) six weeks, and b) 12 weeks.

Figure 4 illustrates the distribution of the RUSHU for the entire cohort based on union outcomes at six and 12 weeks. The median RUSHU for the entire cohort was nine points (IQR 6 to 11) at six weeks and ten points (IQR 8 to 12) at 12 weeks. For those ending up with a nonunion, the median RUSHU was seven points (IQR 5 to 8) at six weeks and eight points (IQR 7 to 9) at 12 weeks. For those achieving fracture union, the median RUSHU was ten points (IQR 8 to 11) at six weeks and 12 points (IQR 11 to 12) at 12 weeks. These differences were statistically significant at both timepoints (p < 0.001, Mann-Whitney U test).

Patients with a RUSHU ≤ eight points were 4.3 times more likely (relative risk (RR) 4.3; 95% CI 2.7 to 6.9) to develop nonunion compared to those with a score of nine points and higher at six-week follow-up. Patients with RUSHU ≤ nine points were 6.1 times more likely (RR 6.1; 95% CI 3.7 to 10.1) compared to those with a score of ten points and higher to develop nonunion at 12 week follow-up. Table III displays statistical parameters indicating the clinical relevance of these cut-off values, such as sensitivity, specificity, positive predictive value (PPV; probability of nonunion), and negative predictive value (NPV; probability of fracture union). Statistical parameters for other cut-off values at six- and 12-week follow-up are available in the Supplementary Tables i and ii.

**Table III.** The statistical properties of the optimal RUSHU cut-off values for predicting nonunion at six and 12 weeks.

Variable	Nonunion	Union	Predictive value
**RUSHU cut-off at 6 weeks (n = 204)**			
8 points or less (n = 100)	66	34	PPV = 0.66
9 points or more (n = 104)	16	88	NPV = 0.85
	Sensitivity = 0.80	Specificity = 0.72	p < 0.001*
**RUSHU cut-off at 12 weeks (n = 206)**			
9 points or less (n = 93)	70	23	PPV = 0.75
10 points or more (n = 113)	14	99	NPV = 0.86
	Sensitivity = 0.83	Specificity = 0.81	p < 0.001*

* Chi-squared test; statistically significant.

NPV, negative predictive value (probability of union if RUSHU is above set threshold); PPV, positive predictive value (probability of nonunion if RUSHU is below set threshold); RUSHU, Radiographic Union Score for HUmeral Fractures.

There were three patients with RUSHU of 11 or 12 points at six weeks and seven patients with RUSHU scores of 11 or 12 points at 12 weeks who eventually developed a nonunion (Supplementary Material radiographs).

### Sensitivity analysis of M cases as two points

At least one cortex was rated “M” in 44 patients at six weeks and in 11 patients at 12 weeks. In the prespecified sensitivity analyses, scoring “M” with two points (instead of three points), the ROC curves yielded slightly lower AUCs of 0.82 (95% CI 0.77 to 0.88) at six weeks and 0.88 (95% CI 0.83 to 0.93) at 12 weeks. The use of various cut-off values did not essentially change the prognostic performance as compared to the primary analysis (Supplementary Tables iii and iv).

### Sensitivity analysis of the FISH trial cohort

The nonunion rate in the FISH trial cohort (28%, 19/69) was noticeably lower than in the total cohort (41%, 92/226). The ROC analysis carried out using the FISH data showed even better discrimination of nonunions compared with the total study cohort with calculated AUCs of 0.90 (95% CI 0.83 to 0.98) at six weeks and 0.97 (95% CI 0.93 to 1.0) at 12 weeks. The top-left corner method provided the best trade-off between sensitivity and specificity, yielding the same cut-off value at six weeks (≤ eight points) and one point higher cut-off at 12 weeks (≤ ten points) compared to the primary analysis. At six weeks, the model yielded 81% sensitivity, 86% specificity, and 65% probability for nonunion in patients scoring eight points or less, and 94% probability for union in patients scoring nine points or more. At 12 weeks, the model yielded 94% sensitivity, 90% specificity, and 77% probability for nonunion in patients scoring ten points or less, and 98% probability for union in patients scoring 11 points or more (Table IV).

**Table IV.** The statistical properties of the optimal RUSHU cut-off values for predicting nonunion in the FISH trial cohort at six and 12 weeks.

Variable	Nonunion	Union	Predictive value
**RUSHU cut-off at 6 weeks (n = 66)**			
8 points or less (n = 20)	13	7	PPV = 0.65
9 points or more (n = 46)	3	43	NPV = 0.94
	Sensitivity = 0.81	Specificity = 0.86	p < 0.001*
**RUSHU cut-off at 12 weeks (n = 68)**			
10 points or less (n = 22)	17	5	PPV = 0.77
11 points or more (n = 46)	1	45	NPV = 0.98
	Sensitivity = 0.94	Specificity = 0.90	p < 0.001*

* Chi-squared test; statistically significant.

FISH, Finnish Shaft of the Humerus; NPV, negative predictive value (probability of union if RUSHU is above set threshold); PPV, positive predictive value (probability of nonunion if RUSHU is below set threshold); RUSHU, Radiographic Union Score for HUmeral Fractures.

## Discussion

We tested the prognostic performance of the RUSHU in a considerably larger cohort than in the original and the previous validation studies, focusing on its reliability, reproducibility, and prognostic accuracy at the six- and 12-week follow-up.

The rationale for developing and using a prognostic tool is to aid healthcare professionals in predicting the likely course or outcome of a medical condition for an individual patient. By stratifying patients based on their risk of certain outcomes, a prognostic tool facilitates shared decision-making: understanding the likely trajectory of a condition aids in the planning and timing of interventions. Prognostic tools guide clinicians in choosing appropriate treatment methods, whether it be aggressive therapeutic approaches for high-risk cases or more conservative strategies for those with a favourable prognosis. However, there are certain requirements for these prognostic tools to be met to justify their use. The assessment of their performance is fundamental to ensuring their reliability and relevance in clinical practice.

Reliability is typically assessed by determining the intra- and interobserver reliability of the tool. To briefly describe the concepts, intraobserver reliability is about consistency within one person, and interobserver reliability is about agreement between different people. In the context of RUSHU, the intraobserver reliability measures how consistently one person can make the scoring when looking at the same radiograph on two separate occasions, while interobserver reliability checks if different observers reach similar scores. Ensuring both are high is important for the reliability and trustworthiness of a prognostic tool. The high intra- and interobserver reliability observed in our study indicate that the RUSHU scoring is likely to be similar when using the tool for the same patient (Table II).

Another important indicator of the performance of a prognostic tool is the ability to differentiate between patients who will and will not experience the outcome of interest. This is usually assessed by determining the discrimination, sensitivity, and specificity of the tool. All three aspects are crucial for evaluating the effectiveness and reliability of a prognostic tool. The discriminatory ability of a prognostic tool is typically determined by calculating the AUC of a ROC curve, which yields a comprehensive measure of a prognostic tool’s discriminative ability. A higher AUC indicates better overall performance.

The sensitivity and specificity of the tool, in turn, focus on the tool’s performance in correctly identifying true positive and true negative cases, respectively. Striking a balance between sensitivity and specificity is essential to avoid false reassurance or undue concern.

Beyond statistical measures, the assessment of the performance of a prognostic tool requires consideration of its clinical utility. This involves evaluating whether its use influences treatment decisions or improves patient outcomes, subsequently enhancing the efficiency of healthcare delivery. In our cohort, the application of the RUSHU improved the prediction of nonunion from the baseline nonunion risk of 41% to 66% at six weeks and 75% at 12 weeks, with the obtained optimal cut-off values (Table III).

As our findings demonstrate, the RUSHU enhances the prediction of nonunion. However, the complex phenomenon of nonunion cannot be adequately captured solely through a simple radiological assessment of bone healing. Accurate prediction of humeral shaft nonunion will likely necessitate the inclusion of various clinical characteristics and patient-related information, in addition to the radiological assessment of bone healing at the fracture site.

### Comparison to previous studies

Our study corroborates prior findings regarding the reproducibility of RUSHU scores. Previous research by Oliver et al,^12^ Fordyce et al,^21^ and Guevel et al^13^ reported interobserver and intraobserver ICCs at six-week follow-up ranging from 0.79 to 0.91 and 0.96 to 0.98, respectively, indicating consistent reliability. However, our study identified a minor disparity, a one-point difference in the cut-off value for predicting nonunion at six weeks compared to the original study. This variance may rise from our use of the closest point to the top-left corner method, whereas the precise method used in the original study remains unspecified. Naturally, a compromise must be found between the test’s sensitivity and specificity to determine the appropriate cut-off. Our findings suggest that a cut-off score of ≤ seven points at six weeks yields 82% specificity but may sacrifice sensitivity, detecting only 63% of nonunions (Supplementary Tables iii and iv).

### Strengths and limitations

The major strength of our study is the size of our cohort, almost as large as all previous studies combined (226 patients compared to 241 patients).^12-14,21^ Our study sample also included patients from a prospective RCT, showing that the RUSHU is a valuable prognostic tool in both a more stringent RCT population (sensitivity analysis) and a larger pragmatic cohort (primary analysis). We also had two independent scorers with a good interobserver reliability and mostly excellent intraobserver reproducibility, and additionally solved the differences between the scorers by a consensus meeting with a minimum of four participants.

One limitation is a probable sampling bias as noted in the methods. In our cohort, the 41% nonunion rate is higher than the expected range of 10% to 33% in everyday clinical practice.^5,7,9,14,22^ This difference can be partly explained by the inclusion criterion, which excluded patients with uneventful healing but no radiological verification of union after 12 weeks. We argue that this made the prediction of nonunion more difficult for the RUSHU by the exclusion of easier-to-score cases that did not require follow-up beyond 12 weeks. This is consistent with the finding that the prognostic performance of the RUSHU improved in our sensitivity analyses that used the FISH trial data only. Another limitation is that the assessment of the callus formation was impaired by non-standardized radiographs in some cases. Specifically, in some lateral projections (26/204 at six weeks and 10/206 at 12 weeks), the visibility of the humeral shaft was obscured by thoracic structures, posing a challenge to accurate evaluation. As suboptimal radiographs are part of normal practice, we did not exclude these cases, but note that most were eventually assessed in a consensus meeting due to differences in the primary scoring.

In conclusion, the RUSHU proves to be a reliable and reproducible radiological scoring system that aids in identifying patients at risk of nonunion at both six and 12 weeks post-injury during nonsurgical treatment of humeral shaft fractures. The statistically optimal cut-off values for predicting nonunion are ≤ eight at six weeks and ≤ nine points at 12 weeks post-injury.

**Take home message**

- The Radiographic Union Score for Humeral Fractures (RUSHU) was shown to reliably and reproducibly identify patients at high risk for nonunion during nonoperative treatment of humeral shaft fractures.

- The statistically optimal cut-off values for predicting nonunion are ≤ eight points at six weeks and ≤ nine points at 12 weeks.

## Data Availability

The datasets generated and analyzed in the current study are not publicly available due to data protection regulations. Access to data is limited to the researchers who have obtained permission for data processing. Further inquiries can be made to the corresponding author.

## References

[1] Sarmiento A Kinman PB Galvin EG Schmitt RH Phillips JG Functional bracing of fractures of the shaft of the humerus J Bone Joint Surg Am 1977 59-A 5 596 601 10.2106/00004623-197759050-00004 873955

[2] Rämö L Sumrein BO Lepola V et al. Effect of surgery vs functional bracing on functional outcome among patients with closed displaced humeral shaft fractures: the FISH randomized clinical trial JAMA 2020 323 18 1792 1801 10.1001/jama.2020.3182 32396179 PMC7218498

[3] Matsunaga FT Tamaoki MJS Matsumoto MH Netto NA Faloppa F Belloti JC Minimally invasive osteosynthesis with a bridge plate versus a functional brace for humeral shaft fractures: a randomized controlled trial J Bone Joint Surg Am 2017 99-A 7 583 592 10.2106/JBJS.16.00628 28375891

[4] Rämö L Paavola M Sumrein BO et al. Outcomes with surgery vs functional bracing for patients with closed, displaced humeral shaft fractures and the need for secondary surgery: a prespecified secondary analysis of the FISH randomized clinical trial JAMA Surg 2021 156 6 1 9 10.1001/jamasurg.2021.0906 33851991 PMC8047733

[5] Toivanen JAK Nieminen J Laine H-J Honkonen SE Järvinen MJ Functional treatment of closed humeral shaft fractures Int Orthop 2005 29 1 10 13 10.1007/s00264-004-0612-8 15611875 PMC3456948

[6] Singhal R Stewart P Charalambous CP A pre-fabricated bracing system for the management of humeral shaft fractures: experience of a centre in the United Kingdom Ortop Traumatol Rehabil 2015 17 5 463 470 10.5604/15093492.1186816 26751746

[7] Harkin FE Large RJ Humeral shaft fractures: union outcomes in a large cohort J Shoulder Elbow Surg 2017 26 11 1881 1888 10.1016/j.jse.2017.07.001 29054684

[8] Serrano R Mir HR Sagi HC et al. Modern results of functional bracing of humeral shaft fractures: a multicenter retrospective analysis J Orthop Trauma 2020 34 4 206 209 10.1097/BOT.0000000000001666 31923040

[9] Ekholm R Tidermark J Törnkvist H Adami J Ponzer S Outcome after closed functional treatment of humeral shaft fractures J Orthop Trauma 2006 20 9 591 596 10.1097/01.bot.0000246466.01287.04 17088659

[10] Whelan DB Bhandari M Stephen D et al. Development of the radiographic union score for tibial fractures for the assessment of tibial fracture healing after intramedullary fixation J Trauma 2010 68 3 629 632 10.1097/TA.0b013e3181a7c16d 19996801

[11] Chiavaras MM Bains S Choudur H et al. The Radiographic Union Score for Hip (RUSH): the use of a checklist to evaluate hip fracture healing improves agreement between radiologists and orthopedic surgeons Skeletal Radiol 2013 42 8 1079 1088 10.1007/s00256-013-1605-8 23564001

[12] Oliver WM Smith TJ Nicholson JA et al. The Radiographic Union Score for HUmeral fractures (RUSHU) predicts humeral shaft nonunion Bone Joint J 2019 101-B 10 1300 1306 10.1302/0301-620X.101B10.BJJ-2019-0304.R1 31564159

[13] Guevel B Gokaraju K Mohamed F Sorensen F Gillott E Domos P A comparative study of 6-week and 12-week Radiographic Union Scores for HUmeral fractures (RUSHU) as a predictor of humeral shaft non-union Shoulder Elbow 2022 14 3 295 303 10.1177/17585732211033154 35599708 PMC9121295

[14] Dekker AP Chuttha S Tambe AA Clark DI Predicting the behavior of humeral shaft fractures: an independent validation study of the radiographic union score for humeral fractures and value of assessing fracture mobility J Orthop Trauma 2021 35 10 555 559 10.1097/BOT.0000000000002063 33480643

[15] Mattila H Keskitalo T Simons T Ibounig T Rämö L Epidemiology of 936 humeral shaft fractures in a large Finnish trauma center J Shoulder Elbow Surg 2023 32 5 e206 e215 10.1016/j.jse.2022.10.020 36435484

[16] Rämö L Taimela S Lepola V Malmivaara A Lähdeoja T Paavola M Open reduction and internal fixation of humeral shaft fractures versus conservative treatment with a functional brace: a study protocol of a randomised controlled trial embedded in a cohort BMJ Open 2017 7 7 e014076 10.1136/bmjopen-2016-014076 28694341 PMC5734401

[17] Bossuyt PM Reitsma JB Bruns DE et al. STARD 2015: an updated list of essential items for reporting diagnostic accuracy studies BMJ 2015 351 h5527 10.1136/bmj.h5527 26511519 PMC4623764

[18] Koo TK Li MY A guideline of selecting and reporting intraclass correlation coefficients for reliability research J Chiropr Med 2016 15 2 155 163 10.1016/j.jcm.2016.02.012 27330520 PMC4913118

[19] Froud R Abel G Using ROC curves to choose minimally important change thresholds when sensitivity and specificity are valued equally: the forgotten lesson of Pythagoras. Theoretical considerations and an example application of change in health status PLoS One 2014 9 12 e114468 10.1371/journal.pone.0114468 25474472 PMC4256421

[20] Mandrekar JN Receiver operating characteristic curve in diagnostic test assessment J Thorac Oncol 2010 5 9 1315 1316 10.1097/JTO.0b013e3181ec173d 20736804

[21] Fordyce W Kennedy G Allen JR et al. Validation of the Radiographic Union Score for Humeral fractures (RUSHU): a retrospective study in an independent centre Shoulder Elbow 2023 15 4 390 397 10.1177/17585732221097092 37538525 PMC10395407

[22] Koch PP Gross DFL Gerber C The results of functional (Sarmiento) bracing of humeral shaft fractures J Shoulder Elbow Surg 2002 11 2 143 150 10.1067/mse.2002.121634 11988725

